# Melatoninergic System in Parkinson's Disease: From Neuroprotection to the Management of Motor and Nonmotor Symptoms

**DOI:** 10.1155/2016/3472032

**Published:** 2016-10-18

**Authors:** Josiel Mileno Mack, Marissa Giovanna Schamne, Tuane Bazanella Sampaio, Renata Aparecida Nedel Pértile, Pedro Augusto Carlos Magno Fernandes, Regina P. Markus, Rui Daniel Prediger

**Affiliations:** ^1^Department of Pharmacology, Centre of Biological Sciences, Universidade Federal de Santa Catarina (UFSC), Campus Universitário, Florianópolis, SC, Brazil; ^2^Queensland Brain Institute, University of Queensland (UQ), Brisbane, QLD, Australia; ^3^Department of Physiology, Institute of Bioscience, University of São Paulo (USP), São Paulo, SP, Brazil

## Abstract

Melatonin is synthesized by several tissues besides the pineal gland, and beyond its regulatory effects in light-dark cycle, melatonin is a hormone with neuroprotective, anti-inflammatory, and antioxidant properties. Melatonin acts as a free-radical scavenger, reducing reactive species and improving mitochondrial homeostasis. Melatonin also regulates the expression of neurotrophins that are involved in the survival of dopaminergic neurons and reduces *α*-synuclein aggregation, thus protecting the dopaminergic system against damage. The unbalance of pineal melatonin synthesis can predispose the organism to inflammatory and neurodegenerative diseases such as Parkinson's disease (PD). The aim of this review is to summarize the knowledge about the potential role of the melatoninergic system in the pathogenesis and treatment of PD. The literature reviewed here indicates that PD is associated with impaired brain expression of melatonin and its receptors MT_1_ and MT_2_. Exogenous melatonin treatment presented an outstanding neuroprotective effect in animal models of PD induced by different toxins, such as 6-hydroxydopamine (6-OHDA), 1-methyl-4-phenyl-1,2,3,6-tetrahydropyridine (MPTP), rotenone, paraquat, and maneb. Despite the neuroprotective effects and the improvement of motor impairments, melatonin also presents the potential to improve nonmotor symptoms commonly experienced by PD patients such as sleep and anxiety disorders, depression, and memory dysfunction.

## 1. Introduction

Parkinson's disease (PD) is a chronic and progressive neurodegenerative disease, characterized by the degeneration of dopaminergic (DAergic) neurons in the* substantia nigra pars compacta* (SNc) and the subsequent decrease of dopamine (DA) levels in the striatum. PD is classically considered a complex motor disorder, related to the pronounced degeneration of the DAergic system, which manifests in bradykinesia, tremor, rigidity, and postural instability. Nowadays, PD is considered a progressive multisystem disease that affects a variety of neurotransmitters, being linked with nonmotor symptoms, such as impaired olfaction and gastrointestinal, genitourinary, cardiovascular, and respiratory dysfunctions, as well as visual, neuropsychiatric, and sleep-related disorder. Contrary to the well-defined motor symptoms, PD nonmotor signals and symptoms are still poorly understood and almost not considered in diagnostic and therapeutic protocols. Recent studies suggest that the nonmotor preclinical phase can span for more than 20 years.

New pharmacological tools for treating PD focus on the relief of symptoms that are observed when the disease is clinically developed. The development of neuroprotective drugs and therapies for impairing the progression or even reducing the extension of the disease needs to rely on the understanding of neuroprotection. Neuroprotection definition is complex and involves the potential for preventing cell death, restoring function of damaged neurons, and increasing neuronal number. The development of drugs to slow or prevent the progression of PD might logically evolve from an improved understanding of the pathogenesis of such disease. In the past few years, certainly there were major advances in this area, improving the prospect for the introduction of neuroprotective therapies. However, despite extensive efforts and research, to date, there is no proven therapy to prevent cell death or to restore affected neurons to a normal state in PD. Preclinical studies in laboratory animals have provided several candidate neuroprotective drugs, but clinical endpoints are readily confounded by any symptomatic effect. Thus, these studies do not provide an unequivocal measure of disease progression that can be used to determine if a drug has a neuroprotective effect.

In this context emerges melatonin, an indolamine synthesized by several tissues such as the pineal gland and by extrapineal sources including macrophages, mast cells, and lymphocytes. Classically, melatonin has been considered the darkness hormone since its synthesis by the pineal gland follows the daily alternation between light and darkness. Melatonin synthesized by immune-competent cells acts in an autocrine or paracrine manner. Melatonin receptors (MT_1_ and MT_2_) are expressed in numerous areas of the central nervous system (CNS). Moreover, the physiological role of melatonin is by far wider than what was considered before including learning and memory, anxiety, depression, and neuroprotection. The present review attempts to provide a comprehensive picture of the role of the melatoninergic system in CNS and to highlight recent findings showing its potential as a new palliative and neuroprotective agent in PD.

## 2. Melatoninergic System

Melatonin was isolated in 1958 by the dermatologist Lerner from 50 grams of lyophilized bovine pineal glands powder [1]. Melatonin was named after its property of lightening the skin of frogs, toads, and fishes. In these early times, Wurtman and collaborators [2] examined the distribution of [^3^H]-melatonin in endocrine and other organs of rats and cats. They showed that iris-choroid, ovary, and periphery organs innervated by the autonomic nervous system, including the heart, take up melatonin [2]. In 1965, a paper authored by Axelrod (Nobel Prize of Medicine, 1970) et al. showed the daily variation of melatonin pineal gland content and proposed that the neural pathway that links the retina to the pineal gland involves sympathetic postganglionic fibres originated in the superior cervical ganglia [3]. In the last years, there is increasing data supporting the concept that melatonin effects are not only due to nighttime pineal gland synthesis but also triggered by melatonin synthesized by extrapineal sources [4, 5].

In mammals, several tissues including the pineal gland, retina [6], macrophages [7, 8], mast cells, lymphocytes [4], human bone marrow cells [9], astrocytes [10–12], and enterochromaffin-like cells found in the gastric mucosa [13], rat oocytes [14], placental trophoblasts [15, 16], and human colostrum phagocytes [17] synthesize melatonin. Melatonin (N-acetyl-5-methoxytryptamine) is synthesized by the acetylation of serotonin and the methylation of N-acetylserotonin [18]. The enzymes aryl-alkyl-N-acetyltransferase (AANAT) and acetylserotonin O-methyltransferase (ASMT), also known as hydroxyl-indole-O-methyltransferase (HIOMT), are highly conserved being detected in unicellular organisms, plants, and animals [19–21].

The pineal gland is an endocrine gland of epithelial origin, as the CNS, formed by pinealocytes (modified neurons), astrocytes, and microglia [18]. Nerve fibres originate from perikarya located in the sympathetic superior cervical ganglion and the parasympathetic sphenopalatine and optic ganglion innervate the gland [22]. Sympathetic activation by darkness is the key inducer of pineal melatonin synthesis. On the other hand, the role of parasympathetic innervation is still not clear. In rats, the muscarinic receptors are present only in the developmental phase [23]. In adult rats, the only cholinergic receptors detected are nicotinic, which leads to a great entrance of calcium ions and the release of glutamate. In turn, glutamate inhibits noradrenaline-induced pinealocytes melatonin synthesis [24]. More recently, the sources and cellular effect of gamma-aminobutyric acid (GABA) were described [25]. Although the cellular and molecular mechanisms involved in the response of these neurotransmitters and hormones are becoming clear, there are little cues regarding their functional role.

In addition, the pineal gland is a circumventricular organ capable of communicating blood signals to the brain through the cerebrospinal fluid [22]. In fact, it reacts to cytokines and to circulating hormones such as glucocorticoids [4, 26]. Pro- and anti-inflammatory mediators modulate pineal melatonin synthesis, adjusting the body to cope with inflammatory responses [26]. Therefore, the unbalance of pineal melatonin synthesis can predispose the organism to inflammatory and neurodegenerative diseases.

The conversion by AANAT of serotonin to N-acetylserotonin (the immediate precursor of melatonin) in pinealocytes is the key event for the daily rhythm of melatonin. Although melatonin is the nocturnal hormone in both diurnal and nocturnal animals, the regulation of its biosynthesis is specific for each one. In diurnal animals, only the activity of the enzyme AANAT is under daily control, while in nocturnal animals both transcription and translation are induced by darkness. AANAT has a very short life span; as soon as it is synthesized, the enzyme is ubiquitinated and targeted to the proteasome, being immediately degraded. This maintains the low level of melatonin during daytime, despite the constitutive synthesis of the enzyme. Darkness transmitted to the hypothalamic suprachiasmatic nuclei by the retinohypothalamic tract activates a polysynaptic pathway, which leads to the release of noradrenaline and adenosine triphosphate (ATP) by the sympathetic nerve terminals that reach the pineal gland [18]. The transcription factor cAMP responsive element-binding (CREB) and the protein kinase A (PKA), activated by cyclic-AMP dependent phosphorylation upon *β*-adrenergic stimuli, induce the transcription of the* Aanat* gene and the phosphorylation of the AANAT enzyme, respectively [18]. The binding of phosphorylated-AANAT to the chaperone 14-3-3 impairs its entry in the proteasome and promotes the conversion of N-acetylserotonin in melatonin by ASMT enzyme [27]. Considering the onset of darkness, the raise in plasma melatonin starts much earlier in diurnal than nocturnal animals. Therefore, in humans, it is possible to detect melatonin as soon as 1 h after darkness, while in rodents at least 3 h is necessary to detect melatonin in the plasma [28, 29].

In the pineal gland, ASMT is regulated by the photoperiod. In Siberian hamsters, that are strict photoperiodic animals, ASMT activity was found to be about two times higher under short photoperiods than under long photoperiods, and the peak of melatonin follows the same profile [30]. In addition, this enzyme was shown to be the limiting step of the biosynthetic pathway [31]. Therefore, while AANAT is the key enzyme for determining the circadian rhythm of melatonin, ASMT is the photoperiodic enzyme; that is, it determines the higher melatonin amplitude in the winter when compared to summer nights.

The presence of melatonin in human appendix mucosa and rat digestive tract was described around 40 years ago. After the demonstration that the enzymes responsible for melatonin synthesis are present in the gastrointestinal tract and that the content of melatonin is maintained even after pinealectomy, it was proposed that melatonin should have a local role, independent of darkness control. Melatonin synthesized in the guts is drained to the liver via hepatic portal vein and may reach concentrations 15 times higher than the blood [32]. This information need to be taken into account due to expanding knowledge regarding the relevance of the gut-brain axis in triggering and management of inflammatory based CNS diseases.

The melatonin synthesized by immune-competent cells is highly relevant for surveillance of biotic and abiotic injuries and stress, as well as for the mounting and resolution of innate immune responses. Melatonin is synthesized by lymphocytes, thymus, spleen, and bone marrow [33–36] and was shown to modify the expression of cytokines and the reactive state of T- and B-lymphocytes [4]. In the last decades, the existence of an immune-pineal axis that considers both pineal and extrapineal productions and also the physiological/pathophysiological state of the organism was proposed [26].

In this context, the central transcription factor in defence responses, the nuclear factor kappa B (NF-*κ*B), orchestrates the role of melatonin by inhibiting or activating its production by different sources [5]. The onset of an acute innate response induced by pathogen- or damage-associated molecular patterns (PAMPs and DAMPs, resp.) turns on/off the melatonin synthesis in a cell-dependent manner. Toll-like receptor 4 (TLR4) activation triggers NF-*κ*B nuclear translocation, decreasing melatonin synthesis in pinealocytes and inducing its production by immune-competent cells [37–39]. The key event for this opposite effect lays on the NF-*κ*B dimer that is preferentially activated in each cell. The NF-*κ*B dimer translocated to the nuclei (p50/p50) in pinealocytes has no transactivating domain (TAD) and impairs* Aanat* transcription [40].

On the other hand, the translocation of a TAD positive NF-*κ*B dimer (RelA/cRel) promotes the gene transcription in immunocompetent cells [41, 42]. The reduction of melatonin during the mounting of an innate immune response increases the expression of adhesion molecules that favour the migration of leukocytes from blood to tissues, playing a role in the inflammatory response mounting [43]. Recently, it was shown that the effect of darkness on endothelial cells primes the expression of microRNAs that negatively regulate cell adhesiveness, inflammatory response, and proliferation [44]. Melatonin synthesized by phagocytes acts in a paracrine manner favouring the phagocytosis of bacteria, fungi, parasites, and cellular debris [17, 41, 45, 46]. The synthesis of melatonin by the pineal gland is restored before the end of the acute inflammatory response, due to the inhibition of NF-*κ*B by glucocorticoids released from the adrenal gland [29, 47, 48].

Melatonin effects are mediated by high affinity G protein coupled receptors (GPCRs) MT_1_ and MT_2_, by nuclear receptors, and by its ability to donate electrons, acting as an antioxidant molecule [49]. The history of melatonin receptors reveals the importance of understanding the kinetic of the radioligands. Experiments with [^3^H]-melatonin did not indicate the presence of high affinity receptors, which were revealed after the use of [^125^I]-2-iodo-melatonin, a molecule that did not cross the plasma membrane [50]. Besides its intrinsic free-radical scavenging characteristics, the antioxidant effect of melatonin may also occur by triggering the MT_1_/MT_2_-dependent induction of enzymes involved in the removal of reactive oxygen and nitrogen species (ROS and RNS), a pathway that requires much lower concentrations of the ligand [4]. Melatonin receptors may also form homo- and heterodimers, increasing the complexity of its pharmacology. Melatonin MT_2_ receptors dimerize with serotonin 5HT_2C_ receptors in transfected human embryonic kidney cells (HEK 293) and human cortical cells [51]. Another interesting observation that could result in future basis for the understanding of melatoninergic drugs effects in health and disease is the dimerization of melatonin receptors with G protein-coupled receptor 50 (GPR50). GPR50 shares similarity with melatonin receptors but does not bind to melatonin [52]. MT_1_ function is blocked by the formation of MT_1_/GPR50 but this effect is not observed when MT_2_ heterodimerizes with GPR50 [53]. Therefore, the results obtained with melatonin agonists and antagonists need to be followed by the understanding of the biology of melatonin receptors molecular complexity.

## 3. Melatoninergic System and Neuroprotection

Neuroprotective mechanisms preserve neuronal structure and function against acute or chronic insults of the CNS. Abrupt changes in brain irrigation by trauma or ischemia or the presence of neurotoxins demand immediate reactions of the nervous system cells. Deleterious effects triggered by endogenous molecules or by dysfunctional processes are the basis of neurodegenerative diseases and brain tumors. Inflammatory changes, ROS and RNS formation, mitochondrial dysfunction, excitotoxicity, apoptosis, and protein aggregation are processes commonly found in these cases and related to neuron injuries [54].

Neuroprotective melatonin effects were shown at the molecular, cellular, and tissue level in animal models and in human trials conducted in patients with different CNS diseases. Reduction of inflammatory mediators, apoptosis, and unbalance of redox state are involved in the neuroprotection induced by melatonin [21, 55–57].

Several studies describe antioxidant effects of melatonin [58, 59]. Indeed, these protective effects are achieved by direct scavenger mechanisms or by melatonin receptors-mediated pathways. Melatonin and the metabolites N1-acetyl-5-methoxykynuramine (AMK) and N1-acetyl-N2-formyl-5-methoxykynuramine (AFMK) are electron donors and scavenge hydroxyl radicals, hydrogen peroxides, and singlet oxygen in a stoichiometric manner [60]. Activation of melatonin MT_1_ and MT_2_ receptors also exerts protective effects, in part dependent on the control of antioxidant enzymes expression such as glutathione peroxidase, glutathione reductase, and superoxide dismutase [61, 62].

The pivotal pathway for triggering inflammatory responses is the uncontrolled activation of the nuclear translocation of NF-*κ*B dimers by PAMPs or DAMPs, promoting neuronal death [37]. Melatonin inhibits the nuclear translocation of NF-*κ*B in several cellular models. Furthermore, melatonin exerts a protective effect by blocking NF-*κ*B activation induced by lipopolysaccharide (LPS) in both rats and cells [63, 64]. NF-*κ*B inhibitors block either the melatonin-induced neuroprotective or neurorestorative effects. Melatonin also inhibits other signalling pathways linked to innate immune responses, as nucleotide-binding oligomerization domain-like receptor family (NLR) pyrine domain-containing 3 (NLRP3) [65, 66]. This receptor located in the inflammasome upregulates apoptotic signalling pathways in the brain and peripheral inflammatory responses. Thus, melatonin impairs the activation of important signalling pathways that are responsible for triggering innate immune response. In the context of the immune-pineal axis, that activation of NF-*κ*B initially inhibits the synthesis of melatonin by the pineal gland, favouring the mounting of the inflammatory response, and as the response progresses, it induces the synthesis of melatonin by microglia [5, 64].

Another interesting link between melatoninergic system and brain inflammatory responses is the regulation of the alpha-7-nicotinic acetylcholine receptors (*α*7nAchR) by melatonin [54, 67–69]. Cholinergic control of inflammation adjusts the intensity of appropriate immune responses and *α*7nAchR activation in immunocompetent cells shifts the immune responses towards a more anti-inflammatory profile. The expression and the functional response of *α*7nAchR located in the sympathetic neurons terminal present a daily variation dependent on the circadian rhythm of melatonin. Moreover, melatonin favours *α*7nAchR expression in the plasma membrane, reducing the desensitization due to internalization [68]. Therefore, melatonin improves *α*7nAchR-dependent cholinergic activities. Indeed, melatonin protects neurons from prion-mediated mitochondrial neurotoxicity by increasing the *α*7nAchR-mediated autophagic flux [54] and ischemic neurons survival by increasing the heme oxygenase-1 (HO-1) expression [69]. Furthermore, melatonin ameliorates cholinergic dysfunction and avoids neuronal apoptosis in the APP 695 transgenic mouse model of AD [67].

Interestingly, neurodegenerative disorders such as AD and PD are associated with impaired brain expression of both melatonin and *α*7nAchR [70–73] and disruption of nocturnal melatonin rhythm [74–76]. It is noteworthy that amyloid *β* (A*β*) peptides inhibit the synthesis of melatonin in rat pineal gland by activating NF-*κ*B [77] and that melatonin treatment improves cognition and sleep in AD patients [78]. Long-term melatonin treatment induces receptor-dependent and receptors-independent neuroprotective effects in animal model of AD [62]. Receptor-independent effects of melatonin were observed on nonspatial cognitive performance, reduction of cortical and hippocampal amyloid plaques, and plasma A*β*_1–42_ levels. On the other hand, melatonin receptor-dependent effects were observed on spatial learning and memory and on the modulation of antioxidant enzymes [62]. Melatonin also attenuated A*β*-induced neurofilament hyperphosphorylation [55] and neuroinflammation [79]. Finally, melatonin restored mitochondrial function in a mouse model of AD via activation of melatonin receptors [56].

Disruption of melatoninergic system in diverse neurodegenerative diseases reinforces the potential neuroprotective effects of melatonin. Huntington's disease (HD) patients present a rhythm delay in nocturnal melatonin production [80]. The clinical relapses in multiple sclerosis are negatively correlated with melatonin levels [81] and no melatonin nocturnal peak is detected in AD [74] and stroke [82] patients. Interestingly, the reduction on nocturnal melatonin production occurs in early stages of AD [75]. Low night levels of melatonin are also commonly reported in schizophrenia patients [83].

The great exception is PD, which shows some controversies in the literature. Some reports refer no alteration [84, 85], small phase alterations [86], or decreased production of melatonin [87]. Nevertheless, Videnovic and colleagues [87] showed that, besides the reduction of circadian melatonin levels in PD, those patients with excessive daytime sleepiness present an even lower production of the hormone. Moreover, nocturnal working was shown to reduce the risk of PD while long daytime sleep was associated with increased occurrence of PD [88, 89].

Alongside the impaired production of melatonin, some neurodegenerative diseases also present altered expression of melatonin receptors. In PD patients, the expression of both MT_1_ and MT_2_ receptors is reduced in the amygdala and SNc [73]. Studies conducted in brains tissues of AD patients showed decreased expression of MT_1_ and MT_2_ in the cortex and in the pineal gland [71], MT_1_ reduction in the suprachiasmatic nucleus [72], and a dual modulation in the hippocampus characterized by increased expression of MT_1_ and decreased expression of MT_2_ [70]. In addition, *β*-amyloid directly binds to MT_1_ and MT_2_ receptors, decreasing MT_1_ binding sites and the activation of downstream pathways triggered by both melatonin receptors subtypes [77].

Finally, it is important to consider the role of extrapineal melatonin synthesized within the CNS as a resilience mechanism against neurodegenerative processes. There are some important considerations regarding how melatonin reaches the CNS, the different capability of each brain area to synthesize melatonin under stressful conditions, and the relevance of melatonin metabolism inside the brain. Although i.p. administration of melatonin may reach the brain [90], daily circadian rhythm of melatonin in the cerebrospinal fluid is due to direct influx of melatonin from the pineal gland (i.e., independent of the plasma concentration) [91–93]. Acute injuries activate the immune-pineal axis blocking melatonin synthesis by the pineal gland and inducing it by immune-competent cells located in the injured area [5, 26]. Injection of LPS in the third ventricle increases AANAT expression in cortex, hippocampus, and cerebellum but induces melatonin synthesis only in the cerebellum [64]. This effect is mediated by the activation of NF-*κ*B pathway and it was observed that cerebellar, but not cortical and hippocampal, neurons present reduced number of neuronal deaths after LPS injection. However, when LPS was given after the blockade of melatonin MT_1_/MT_2_ receptors with the nonselective competitive antagonist luzindole, there was a significant increase in cerebellar neuronal death.

Therefore, this review will assess the role of melatoninergic system in PD, focusing on the antioxidant, anti-inflammatory, motor, and nonmotor effects of melatonin. Besides the protective effects of melatonin described in the next sessions, a question that still has no answer is whether a putative local production of melatonin by some patients would explain the contradictory observations aforementioned regarding the nocturnal melatonin production in PD patients.

## 4. Parkinson's Disease

PD is characterized by a slow and progressive loss of DAergic neurons in the SNc, an important pathway for fine modulation of motor function [94]. The first report of PD is dated in 1817 in the James Parkinson's monograph “An Essay on the Shaking Palsy.” Symptoms observed by Parkinson were described as “involuntary tremulous motion with lessened muscular power, in parts not in action even when supported, with a propensity to bend the trunk forward and to pass from walking to a running pace” [95]. Since then, PD is known as a movement disorder that mainly affects people of middle to old age [96].

PD is highly linked with the aging, being responsible for a significant increase in morbidity and mortality, affecting the social and economic life of individuals and society [57, 97]. According to the United Nations, it is believed that the number of elderly people in the world will reach 2 billion in 2050. Due to this, the World Health Organization (WHO) accounts that the number of cases of PD tends to increase and the importance of the disease as a public health problem will become even more significant. Additionally, PD is more common in males, with a ratio of men to women of 3 : 2 [98].

Although the symptoms and the neuropathology of PD have been well characterized, the mechanisms and causes of the disease remain unclear. Therefore, PD is described as a multifactorial disease that could be influenced by age and genetic and environmental factors [99–101]. PD is responsible for a significant increase in morbidity and mortality, affecting the social and economic life of individuals and society [57, 97].

The environmental hypothesis of PD aetiology is based on epidemiological studies showing the high frequency of the disease in individuals exposure to xenobiotics such as agricultural chemicals (pesticides, herbicides, etc.) and heavy metals (iron, manganese, aluminium, etc.) [102]. Other hypotheses of PD aetiology include genetic factors, natural aging process, increase of the formation of reactive species or inadequate functioning of its neutralizing mechanisms, and reduction of ATP stores that end up leading to neuronal death [103].

The majority of cases (90–95% of cases) are idiopathic, affecting 5% of population over 85 years old. However, in 5–10% of cases, PD can have a genetic component showing recessive and/or dominant modes of inheritance. Several genes mutations have been identified resulting in PD. Early onset PD or familial PD (occurring in people under 50 years of age) is less common, and recent data has identified some nuclear genes associated with familial PD, like* LRRK2*,* PARK2*,* SNCA*,* PARK7,* and* PINK1* [104].

Despite the knowledge derived from genetic research in PD, the accurate mechanism underlying the DAergic loss in PD is still not understood. However, mitochondrial dysfunction and oxidative stress in which there is an increase of the production of ROS, abnormal protein handling, neuroinflammation, excitotoxicity, and apoptotic processes have a central role in PD pathogenesis [101, 103].

The neuron's environment is a main contributor to neurodegeneration. Many evidences suggest that neurodegeneration can occur because of a cascade of events that affects the neuron's environment, called neuroinflammation [105]. Neuroinflammatory mechanisms involved in PD pathogenesis comprise microglial activation, astrogliosis, and lymphocytic infiltration and also involve alterations in the morphology of glial cells, including both astrocytes and microglia, indicating their interactions with the alterations in the homeostasis of the environment [101, 105]. Neurodegeneration in PD may be accompanied by an inflammatory reaction, characterized by activation of microglia, which leads to production of a number of inflammatory mediators (e.g., NF-*κ*B, interleukin-1 (IL-1), IL-6, IL-1*β*, cyclooxygenase-2 (COX-2), tumoral necrosis factor-*α* (TNF-*α*), inducible nitric oxide synthase (iNOS), interferon-*γ*) and increased expression of different proinflammatory cytokines by glial cells. Besides, an increase in the level of these components in the substantia nigra and in the cerebrospinal fluid (CSF) and an elevation of *γ*/*δ* + T cells in the peripheral blood and CSF of patients with PD were also reported [101, 106, 107]. This increase in the proinflammatory cytokine levels could switch on different apoptotic pathways involved in the degeneration of DAergic neurons [94]. Taken together, oxidative stress, accumulation of endogenous cell metabolism products, excitotoxins like glutamate, toxic proteins, and a decrease of trophic factors can lead to apoptotic events of neurodegeneration [108, 109].

Oxidative stress also plays a pivotal role in DAergic neurodegeneration in PD. The DAergic neurons themselves seem to contribute to ROS production through DA metabolism, which produces superoxide anion, hydroxyl radical, and hydrogen peroxide. Furthermore, the autoxidation of DA produces DA-quinone, a molecule that can damage the proteins structure [109].

Reactive species are involved in the protein aggregation. In PD, the presence of *α*-synuclein and ubiquitin aggregates in neurons in processes of degeneration is observed, especially in the DAergic neurons from SNc. These aggregates, known as Lewy bodies, are pathological markers for PD, being found in 85% of autopsies of PD patients [110, 111].

*α*-Synuclein plays an important role in synapse maturation and maintenance of neurons and its expression is developmentally regulated, redistributing from the cell bodies to synaptic terminals during periods of neuronal differentiation [112]. Also, this protein is upregulated during periods of synaptic plasticity [112]. Studies suggest that the *α*-synuclein can regulate the function of the two key proteins that control the amount of DA inside nerve terminals, regulating the uptake of extracellular DA by the DA transporter (DAT) and the packaging of cytosolic DA by the vesicular monoamine transporter 2 (VMAT2) [112]. Thus, the aggregation of *α*-synuclein triggers cellular mechanisms that lead to progressive death of DAergic neurons [113].

Many environmental toxicants, in particular pesticides, have been considered risk factors for PD. There are many studies evaluating the influence of exposure to heavy metals, solvents, and carbon monoxide in PD development [99, 114, 115]. Epidemiological studies have pointed a clear association between increased PD risk and specific environmental factors such as rural residence, drinking water from wells, and exposure to agricultural chemicals, including paraquat, rotenone, maneb, dieldrin, fungicides, and organochlorines [99, 102, 116, 117].

The degeneration of DAergic neurons in PD occurs mainly in the basal ganglia, a group of subcortical nuclei that control the voluntary movements (besides many other functions including procedural memory processing). The basal ganglia is composed of the striatum (putamen and caudate), globus pallidus externa and interna, subthalamic nucleus, SNc, and the substantia nigra pars reticulata (SNr). The basal ganglia forms neural loops extending from motor cortex to the motor thalamus and back to the cortex, and these loops seem to control the movements at a varying level of complexity [96]. The degeneration of DAergic neurons in PD occurs mainly in the nigrostriatal pathway and in minor extension in the ventral tegmental area [96].

Motor symptoms of PD begin to manifest when approximately 70% of DAergic neurons in the nigrostriatal pathway have been lost. In the beginning of the disease, the motor symptoms can be unnoticed or misinterpreted for a long time. Fatigue, lugubrious stiff face, impairment in the quality of speech, and extreme slowing down can be the first signals of PD and sometimes they can be confused with other diseases or with the aging itself [104]. The classical motor symptoms of PD include resting tremor, bradykinesia, muscular rigidity, and postural imbalance [118].

Additionally, a growing body of evidence demonstrates that PD patients commonly present sleep disturbances [87, 119, 120], since DA is a neurotransmitter of major importance for the circadian cycle. Sleep disturbances are among the most common nonmotor symptoms of PD, affecting about 90% of PD patients. Impaired circadian function in PD is related not only to the disruption of sleep-wake cycle, but also to other functions such as autonomic imbalance, cognitive decline, and psychiatric disorders [121, 122].

Nowadays, it is well known that other neurotransmitter systems (e.g., cholinergic, noradrenergic, and serotonergic) also degenerate in PD, and the cell loss is extended to other brain regions besides the nigrostriatal pathway. This non-DAergic degeneration is a major cause of the nonmotor symptoms of PD such as cognitive decline, mood disorders, and autonomic dysfunctions. Nonmotor symptoms of PD are present across all the disease stages, but the frequency and severity increase with disease progression. Many studies have shown that these symptoms are highly incapacitating and have a major impact on the quality of life ahead of motor symptoms [123].

Besides the sleep disturbances, nonmotor symptoms of PD also include cognitive decline, depression, and anxiety [124, 125]. Most of studies demonstrated that the psychiatric symptoms are related to monoaminergic deficits within components of the limbic system implicated in emotional and affective functions [125]. As demonstrated by Braak et al. [126], the progressive death of DAergic neurons in PD occurs alongside the degeneration of noradrenergic and serotonergic systems.

The current available drugs for PD (see Table 1) mainly improve motor symptoms, without preventing the progression of the disease. Besides, the available treatments can cause several motor complications following chronic use [127] and eventually these treatments present low efficacy [128].

The most effective treatment for PD involves DA replacement therapy, made by the administration of its precursor 3,4-dihydroxy-L-phenylalanine (L-DOPA) associated with dopa-decarboxylase inhibitors (benserazide or carbidopa). Although such treatment improves motor symptoms of PD, the long-term treatment with L-DOPA is inefficient and causes numerous complications [129].

Aside from the treatment with L-DOPA, there are new treatment strategies focusing on the constant stimulation of the DAergic system, for example, the use of drugs with longer half-lives such as the DA receptor agonists ropinirole and pramipexole that allows the later use of L-DOPA or other drugs with short half-life [100, 130]. Table 1 summarizes the drugs available for symptomatic treatment of PD.

Despite all these options for PD treatment, none of these drugs prevent the disease progression. Therefore, there is a need to develop drugs or interventions that prevent or slow the progression of the degeneration of DAergic and non-DAergic neurons in PD.

## 5. Neuroprotective Potential of Melatoninergic System in Parkinson's Disease

Several studies have shown that PD patients exhibit changes in the melatonin production and in the expression of melatoninergic receptors MT_1_ and MT_2_ in the SNc [73, 84–87]. This reduction in endogenous melatonin production in PD patients, along with the discovery of the antioxidant activity of melatonin, has led to increasing interest in affording neuroprotection in PD. In this regard, it is known that the CNS is highly vulnerable to the effects of ROS, mainly due to high consumption of oxygen from this tissue, and that oxidative stress has a significant importance in the pathogenesis of PD [183].

**Figure 1:**
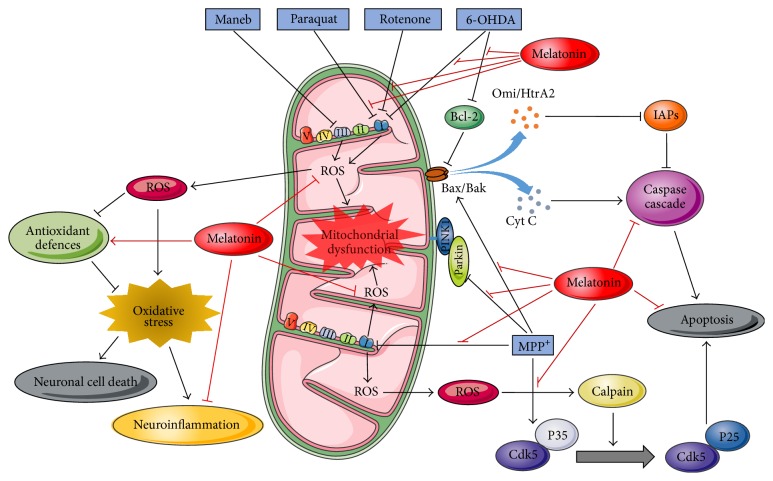
Summary of molecular mechanisms associated with neuroprotective effects of melatonin in* in vivo* and* in vitro* models of Parkinson's disease. The main molecular mechanism of neurotoxins is related to its ability to inhibit the complexes of the mitochondrial electron transport chain. The inhibition of these complexes leads to an increased production of reactive oxygen species (ROS) and, consequently, to mitochondrial dysfunction, oxidative stress, activation of apoptotic pathways, and neuroinflammation, culminating in neuronal cell death. Melatonin exerts neuroprotective effects through different mechanisms: protection of the complex I activity, neutralization of ROS, increased cell antioxidant defences, reducing neuroinflammation, inhibition of caspases cascade, and cellular apoptosis. Melatonin is also able to protect against induction of Bax and Cdk5/p35 expression and inhibition of Parkin/PINK1 and Bcl-2 expression induced by toxins in PD models. 6-OHDA: 6-hydroxydopamine; Bak: Bcl2 antagonist/killer; Bax: Bcl2 associated X; Bcl2: B cell leukemia/lymphoma 2; Cdk5: cyclin-dependent kinase 5; Cyt C: Cytochrome C; IAPs: inhibitors of apoptosis proteins; MPP^+^: 1-methyl-4-phenylpyridinium; Omi/HtrA2: HtrA serine peptidase 2; ROS: reactive oxygen species.

Diverse neurotoxins have been used to mimic behavioural and neurochemical characteristics of PD in laboratory animals and thereby improve the knowledge about the pathogenesis and molecular mechanisms of the disease (Figure 1). These neurotoxins-based models are also useful for the screening of potential new treatments, including new agents aiming neuroprotection [184]. In this context, exogenous melatonin administration has demonstrated an outstanding neuroprotective effect in animal models of PD induced by different toxins such as 6-hydroxydopamine (6-OHDA), 1-methyl-4-phenyl,1-1,2,3,6-tetrahydropyridine (MPTP), rotenone, paraquat, and maneb (Table 2) [132, 144, 161, 164].

6-OHDA was the first toxin used to model the DAergic neurodegeneration similar to that seen in PD [185]. In DAergic neurons, 6-OHDA is internalized via DAT, reaching the interior of the cell where it will elicit its toxic effects [186]. The 6-OHDA exerts its toxicity mainly through two mechanisms: (1) autooxidation and (2) the inhibition of complexes I and IV of the mitochondrial electron transport chain. These mechanisms increase ROS production, which can induce neuroinflammation, microglial activation, and induction of apoptotic pathways culminating in cell death [186].

Unilateral nigrostriatal lesions induced by 6-OHDA followed by challenge with DA receptor agonists (e.g., apomorphine) lead to rotational behaviour in these animals; the rotational behaviour is mainly caused by the increased sensitivity to DA as a consequence of upregulation of DA receptors, in response to the reduced release of this neurotransmitter in the striatum [187]. Melatonin treatment confers DAergic neuroprotection through the normalization of oxidative unbalance generated by 6-OHDA administration, which was characterized by the measurement of expression and activity of antioxidant enzymes and levels of lipid peroxidation [132, 133, 143]. This effect may be due to the ability of melatonin to neutralize reactive species [188] or by the melatonin-induced increased activity [143] and expression of antioxidant enzymes [132]. Furthermore, Dabbeni-Sala et al. [135] demonstrated that melatonin is able to protect the 6-OHDA-induced inhibition of complex I activity of the mitochondrial electron transport chain in mice. In addition, melatonin also led to c-Jun phosphorylation inhibition, increased Bcl-2 levels, and decreased caspase-3 activity, blocking the apoptosis induced by 6-OHDA [132, 134, 137, 141] (Table 2).

The anti-inflammatory effect of melatonin was also observed in 6-OHDA model of PD. Melatonin inhibited COX enzyme activity and reduced the prostaglandin E2 levels (PGE2) [141]. Of high importance is the notion that the potential neuroprotective effects of melatonin in PD were demonstrated by independent research groups, where melatonin protected against the 6-OHDA-induced loss of tyrosine hydroxylase (TH) positive neurons in the SNc and striatal projections accompanied by significant improvement of motor impairments in rodents [131, 135, 136] (Table 2).

MPTP is another toxin widely used to mimic PD in animal models. This toxin was first described by Langston et al. [189] after causing a permanent Parkinsonism in drug users of northwestern California. The MPTP was accidentally produced during the synthesis of 1-methyl-4-phenyl-4-propion piperidine (MPPP). After administration, MPTP crosses the blood-brain barrier and is converted into the active toxin 1-methyl-4-phenylpyridinium (MPP^+^) by the enzyme monoamine oxidase B (MAO-B) present on glial cells. MPP^+^ has high selectivity for DAergic neurons since it is internalized by the DAT present in these cells. Inside the cell, MPP^+^ inhibits the activity of complex I of the mitochondrial electron transport chain, causing an increase in the production of free radicals. This effect leads to activation of apoptotic pathways and consequent death of DAergic neurons [190]. MPTP studies often use systemic administration of this toxin or infusion of MPP^+^ directly into the target structure, because MPP^+^ does not cross the blood-brain barrier. Melatonin treatment prevented the loss of TH-positive neurons in the SNc at the same time which improved motor deficits induced by MPTP [144, 151]. Furthermore, melatonin, when coadministered with L-DOPA, was able to improve the motor benefits induced by L-DOPA [157] (Table 2).

The neuroprotective effect of melatonin seems to be mainly related to the its antioxidant activity, promoting reduced levels of lipid peroxidation, free radicals, oxidative damage in the mitochondrial DNA, and protection of the levels of GSH and antioxidant enzymes activity that are impaired by MPTP/MPP^+^ administration [147, 151, 153, 155, 156] (Figure 1). Further, Khaldy et al. [191] showed that melatonin was able to prevent hydroxyl radical generation evoked by DA autoxidation evaluated* in vitro*. The mechanisms of melatonin neuroprotection (summarized in Figure 1) were investigated in* in vitro* and* in vivo* studies.

Some other mechanisms of melatonin neuroprotection have been demonstrated. Neurotrophic factors are important for the development, maintenance, and function of neurons and glial cells [192]. Glial cell line-derived neurotrophic factor (GDNF) is a neurotrophin known to promote the survival of DAergic neurons. GDNF expression was increased in rat C6 glioma cells exposed to melatonin [193]. The C17.2 neural stem cell line expresses MT_1_ receptors and melatonin was also shown to increase GDNF expression in these cells [194]. Systemic MPTP administration in rats during 7 days decreased DA levels and increased GDNF mRNA expression in the striatum, while intrastriatal injections of melatonin further enhanced GDNF mRNA expression in this brain structure [195].

Another important neurotrophin that has potent effect in the survival and morphology of DAergic neurons is the brain-derived neurotrophic factor (BDNF) [196]. Howells and colleagues showed that BDNF expression is reduced in the SNc of PD patients [196]. However, nM concentrations of melatonin were shown to increase BDNF levels and exert neuroprotective effect in MT_2_-knockout mice and in mouse cerebellar granule cells that underwent low-potassium toxic insults [197]. Rats tested under sleep deprivation and treated with melatonin also showed increased levels of BDNF in the cerebral cortex and hippocampus [198]. In addition, BDNF mRNA levels were increased in rat hippocampus and prefrontal cortex after acute administration of the MT_1_/MT_2_ agonist agomelatine [199]. Therefore, the regulation of the expression of neurotrophic factors by melatonin is likely part of the mechanism of neuroprotection exerted by melatonin.

Given the importance of *α*-synuclein alterations in PD, some studies have focused on the effect of melatonin in *α*-synuclein expression and aggregation.* In vitro* studies showed that melatonin protects dopaminergic cells such as SK-N-SH from the neurotoxicity induced by amphetamine (AMPH) and prevents the toxic overexpression of *α*-synuclein that occurs when these cells are exposed to AMPH [110, 200]. In this cell model, the AMPH increases *α*-synuclein expression while reducing TH phosphorylation, which is necessary for TH activation and DA synthesis [200]. An* in vivo* study by Sae-Ung et al. [201] revealed that subcutaneous injections of AMPH in rats significantly increased *α*-synuclein levels in the SNc, nucleus accumbens, striatum, and prefrontal cortex. However, the concomitant administration of AMPH and melatonin drastically reduced *α*-synuclein accumulation [201]. In a model of kainic acid-induced neurotoxicity in C57BL/6 mice, the hippocampal *α*-synuclein aggregation was reduced by the oral administration of melatonin 1 h prior to kainic acid injection [202]. Taken together, these results demonstrate the potential of melatonin to modulate *α*-synuclein expression and protect DAergic cells against its undesirable toxic alterations (Table 2).

Melatonin also has a protective effect on the mitochondria, preventing the MPTP-induced inhibition of the complex I activity of the electron transport chain and the stimulation of iNOS activity, avoiding the potential collapse of mitochondrial membrane triggered by MPTP [145, 147, 153]. Neuroinflammation evoked by MPTP administration, characterized by increased levels of inflammatory proteins, mRNA proinflammatory cytokines, and NF-*κ*B, was also attenuated by melatonin treatment [154] (Table 2).

The mechanism of MPTP toxicity appears to involve the stimulation of the phosphorylation of p38-MAPK, increasing the cdk5-35 expression and its cleavage to cdk5-25, which are involved in neurodegeneration [148, 154]. The administration of MPTP/MPP^+^ also leads to a reduction in the mRNA and expression of fibroblast growth factor 9 (FGF9), which participates in the proliferation, differentiation, and survival of the cell. The expression and function of* parkin/PINK1/DJ-1/MUL1* loop, which plays an important role in maintaining mitochondrial homeostasis, are also altered by this toxin [150, 158]. All these changes were normalized by melatonin treatment [148, 150, 154, 158] (Table 2).

As described before, epidemiological studies have shown that exposure to certain pesticides increases the risk of developing PD and this has led to a growing interest in the development of animal models using environmental toxins such as rotenone, paraquat, and maneb [203]. Rotenone can exert its toxic effects by inhibiting the activity of complex I of the electron transport chain in mitochondria, leading to generation of ROS. Similar to MPTP, rotenone can be administered systemically or directly into the target brain structure of rodents and in cell cultures [186]. Melatonin has a protective effect against neurodegeneration caused by rotenone in different experimental protocols. Melatonin treatment reduced oxidative stress caused by rotenone, which was evidenced by reduced levels of hydroxyl radicals in the mitochondria, protection of GSH levels, and activity of antioxidant enzymes [160]. Melatonin also inhibited apoptosis induced by rotenone by reducing the Bax expression and release of Omi/HtrA2 [204]. This neuroprotective effect of melatonin culminated in the prevention of motor deficits evoked by rotenone administration in rodents and in a Parkinsonism model in* Drosophila melanogaster* [159, 161, 162] (Table 2).

Maneb and paraquat are pesticides used to mimic PD in cells and animals and they can be administered alone or in combination, which increases their toxicity. Paraquat and maneb can inhibit the biosynthesis of ATP and induce the formation of ROS by inhibiting the activity of complexes I and III of the mitochondrial electron transport chain, respectively [186]. Melatonin showed neuroprotective effect against toxicity induced by these pesticides through inhibition of oxidative stress and apoptotic pathways [163, 164]. In addition, melatonin was able to inhibit the aggregation of *α*-synuclein in P12 cells exposed to maneb [163] (Table 2).

In summary, melatonin demonstrated neuroprotective effects in different experimental models of PD. This neuroprotection seems to be mainly dependent on the modulation of the redox state of the cell by the melatonin [133, 143, 144, 147, 151, 153, 164]. However, the models currently used to mimic the PD have limitations and do not accurately correspond to the disease in humans. In addition, the adverse effects of chronic administration of melatonin are still poorly understood. Thus, further studies using genetic models of PD are necessary to confirm the neuroprotective potential of melatonin in the disease.

## 6. Melatoninergic System as a Putative Target for Treating Motor and Nonmotor Symptoms of Parkinson's Disease

### 6.1. Motor Symptoms

Many studies have assessed the possible role of melatonin in the modulation of motor symptoms of PD with discrepant findings [205]. The majority of studies showing the benefits of melatonin on evoked motor deficits in animal models of PD performed a pretreatment with melatonin. In such studies, melatonin exerts neuroprotective effects by preventing the death of DAergic neurons in the SNc and thus avoiding the development of motor dysfunction. However, Gutierrez-Valdez et al. [140] employed a different protocol where rats underwent a unilateral injection of 6-OHDA into the medial forebrain bundle and were treated with melatonin or L-DOPA, 4 days after the injury. Abnormal irregular movements (AIMs) evaluation, and the beam walking test were performed to assess dyskinesia and motor function. Melatonin was able to improve the rats walking ability without inducing the onset of AIMs, as in the case of L-DOPA treatment. Furthermore, melatonin treatment attenuated the loss of TH-positive neurons and protected the ultrastructural preservation of striatal neurons. These results suggest that although melatonin treatment started 4 days after the lesion, the beneficial effects on the motor symptoms were maintained. Other studies with PD models using 6-OHDA and MPTP also showed that treatment with melatonin, initiated shortly after administration of toxins, inhibited the onset of motor deficits. However, this effect seems to be related to the neuroprotective ability of melatonin instead of a direct action on the DAergic transmission [135, 136, 138, 156].

On the other hand, other studies failed to observe motor benefits of melatonin treatment in animal models of PD or PD patients. Medeiros et al. [206] showed that melatonin administration (3 mg, 1 h before bedtime) for 4 weeks in PD patients was able to improve the quality of sleep but did not affect motor symptoms in these patients. However, the evaluation of the melatonin effect was carried out during the day without analysis of the acute effect of melatonin. Yildirim and collaborators [141] evaluated the effects of pre- and posttreatment with melatonin in animals that underwent a unilateral lesion with 6-OHDA in locomotor parameters, COX and caspase-3 activity, PGE2 and nitrite/nitrite levels, and apoptosis. Even though melatonin administration was able to prevent/recover parameters related to neuroinflammation and apoptosis, the pre- and the posttreatment were not able to restore the motor deficits induced by 6-OHDA injection. Moreover, Bassani et al. [161] reported that the melatonin posttreatment for 28 days preserved the TH-positive neurons and DA levels of rotenone-lesioned rats. In addition, they also observed that melatonin improved the depressive-like behaviour, but not locomotor deficits, of these animals.

Notably, the administration of melatonin* per se* has been linked to a reduction in locomotor activity in naïve animals. For instance, Chuang and Lin [207] demonstrated that the administration of high melatonin doses (60 mg/kg) led to a reduction in spontaneous locomotor activity of mice under normal conditions and also in response to thermal stress (cold, 4°C, and heat, 36°C). However, these changes were not observed with a lower dose of melatonin (30 mg/kg). Melatonin-treated animals showed a reduction in serotonin release in the hypothalamus, striatum, and nucleus accumbens, although DA levels were not altered. Furthermore, Willis and Armstrong [208] evaluated the effects of slow melatonin release via intracerebroventricular implants, pinealectomy, or exposure to constant light on the motor function of rats submitted to 6-OHDA or MPTP administration. Melatonin administration deteriorated motor performance, exacerbating the deficits evoked by these neurotoxins. Additionally, the experimental antagonism achieved by pinealectomy or melatonin synthesis inhibition by the exposure to light improved the induced motor deficits in PD models [208].

In this regard, previous studies have shown that light therapy may have a beneficial effect in motor and nonmotor symptoms of PD [209]. The improvement of motor symptoms by light was observed in animals with unilateral lesion with 6-OHDA, or with mechanical injury of the lateral hypothalamus [205]. Light therapy was also shown to be beneficial in PD patients. In a clinical study, 20 patients with PD received light therapy by exposure to fluorescent light for 1–1.5 hours at an intensity ranging between 1000 and 1500 lux, about 1 hour before bedtime. Several behavioural parameters were evaluated before the light therapy and after 2 and 5 weeks, at regular intervals.

Light therapy improved rigidity and bradykinesia, improved mood and sleep, reduced seborrhoea and impotence, and increased appetite [210]. In another study, 36 patients with PD received light therapy. The treatment consisted of exposure to 7500 lux illumination in the treated group and 950 lux in the placebo group, 30 min/day for 15 days. Patients were evaluated for motor symptoms, depression, and sleep disorders. The results revealed a significant improvement in tremors and depression presented by these patients [211]. Willis and colleagues [205] conducted a retrospective study where 129 PD patients were monitored for periods ranging from a few months to eight years. The treatment consisted of 1 h of exposure to a light intensity ranging from 4000 lux to 6000 lux before bedtime. Motor and nonmotor symptoms were monitored. Patients were divided into 3 groups: patients who adhered, patients who adhered partially, and patients who did not adhere to the treatment. Patients who adhered to the treatment demonstrated an improvement of bradykinesia, rigidity, and balance. Symptoms of sleep disorders and depression also showed improvement. Additionally, increased medication was also lower in patients that fully or partially adhered in comparison to those who did not adhere.

Other studies also investigated the effects of melatonin receptors antagonism in animal models of PD. For instance, it was demonstrated that ML-23, an antagonist of melatonin receptors, enhanced the motor deficits induced by MPTP [212] and 6-OHDA [213]. This effect may be related to the interaction between the nigrostriatal and retinohypothalamic circuits, which converge towards the lateral hypothalamus. Additionally, the presence of visual impairments related to dopaminergic degeneration in patients with PD reinforces this concept [214].

The beneficial effects of melatonin antagonism on motor symptoms of PD can be explained by the melatonin action in the inhibition of DA release in various brain structures. This inhibition can occur at concentrations ranging from nM to *μ*M and may be a consequence of the inhibition of calcium influx into neuronal terminals [215]. Studies have shown that DA metabolites exhibit a circadian variation with higher concentrations during the day and a reduction in the evening [216]. Khaldy et al. [217] demonstrated that the peak of melatonin release in C57BL/6 mice coincided with a reduction of DA levels in striatum. Moreover, this circadian cycle was disrupted by pinealectomy and restored by melatonin treatment [217]. The striatal DA release also modulates the activation of NMDA receptors. It has been proposed that the inhibition of striatal DA release by melatonin could also reduce striatal neurons response to glutamate released by cortical afferents [215].

In summary, although melatonin has shown a potential in improving motor deficits in animal models of PD, these effects appear to be related to its neuroprotective capacity. Nevertheless, in animal models and PD patients with established DAergic damage, melatonin may exacerbate motor deficits [205]. Moreover, the antagonism of melatonin receptors may lead to improvement in motor symptoms [208, 213]. Further studies are necessary to better understand the balance between DA and melatonin levels in the brain as well as its role in control of motor function.

### 6.2. Nonmotor Symptoms

#### 6.2.1. Sleep Disturbances

Sleep disorders have been increasingly associated with PD and can arise as a prodromal symptom appearing years before the motor symptoms. These disorders may also reach the patient after the PD diagnosis worsening the life quality of patients and caregivers. The appearance of these disorders may be related to the degeneration of neural structures related to the sleep control. Among the sleep disorders that precede or appear in advanced stages of PD are excessive daytime sleepiness (EDS), rapid eye movement (REM), sleep behaviour disorder (RBD), and insomnia [122, 218].

EDS is characterized as a chronic condition in which the individual is not able to remain awake during the day, inducing social problems and automobile accidents. Diagnosis can be performed with the use of a questionnaire such as Epworth Sleepiness Scale [218]. Studies have shown increased risk of PD in patients with EDS [87, 89]. Abbott and colleagues [219] showed an odds ratio of 3.3 for the development of PD in EDS patients monitored from 1994 to 2001. In this regard, the increase of daytime napping also has been associated with a major odds ratio of PD development in 3 different stages: established PD, recent PD, and prediagnosed PD [89]. Although there is some disagreement in the literature, the early onset of EDS has been established as a preclinical and premotor marker of PD.

The EDS may be present in PD as a persistent state of sleepiness or “sleep attacks” in which the patient begins to sleep suddenly. Although studies about the prevalence of EDS in PD show a large variation [218], its frequency appears to increase with disease progression. Tholfsen and colleagues [220] evaluated the frequency of EDS in drug-naive PD patients and after 1, 3, and 5 years of medication. EDS frequency increased from 11.8% at the baseline to 23.4% after 5 years in these patients. This elevation can be related to progressive loss of mesolimbic DAergic neurons and other non-DAergic neuronal circuits that modulate the sleep [218]. However, DAergic drugs used in PD treatment such as L-DOPA and DA receptor agonists may have sedative effects, contributing to the occurrence of EDS [221].

Changes in nocturnal sleep period may or may not be correlated with the appearance of EDS, which explains the variability in the response to the treatments that enhance nighttime sleep. Some pharmacological treatments for EDS have been tested such as modafinil, caffeine, and amphetamine [221]. Modafinil is the most studied drug for this use; however, it shows modest effects on subjective parameters. Its mechanism of action is not completely understood but evidences suggest a link with the modulation of hypothalamic sleep-wake system. Modafinil promotes an increase in wakefulness by stimulating the tuberomammillary nucleus activity and reducing the promotion of sleep exerted by the ventrolateral preoptic area [221]. Caffeine did not provide benefits to sleepiness, but the patients displayed an improvement in motor symptoms of PD. Methylphenidate is an amphetamine that has shown some effect in reducing fatigue in PD patients; however, this drug has a high potential for the development of abuse [221].

The light treatment has been associated with improvement of the EDS symptoms. Mishima et al. [222] demonstrated that EDS was associated with the appearance of changes in melatonin levels and that the application of bright light therapy produced significant enhancements in symptoms. However, no study has evaluated the effect of light therapy in EDS presented in PD patients.

The relation between melatonin levels and EDS onset in patients with PD is still poorly explored. The cross-sectional study conducted by Videnovic and colleagues [87] with PD patients regularly receiving DAergic therapy revealed a diminished melatonin release. In addition, PD patients with EDS exhibited a lower release of melatonin than PD patients without EDS. Nevertheless, exogenous melatonin was not effective on EDS [223].

RBD is a parasomnia whose main features are the nocturnal dream enactment behaviour and loss of atonia of skeletal muscles. Abnormal motor behaviour (kicking) and vocals (screaming, laughing, and crying) also are commonly found in patients with this disorder. The motor symptoms provide fracture risk for the patient and their bed partner. The magnocellular nucleus in the medulla and subcoeruleus locus, which are related to the control of atony, appears to be altered in RBD. The appearance of RBD has been strongly linked to an increased risk of PD [121, 218, 224]. Schenck et al. [225] found that 38% of RBD patients evaluated in their study also have PD. In agreement, Iranzo and colleagues [226] observed that, of 44 patients diagnosed with idiopathic RBD (IRBD), 81% (36 patients) presented some degenerative syndrome, among them 16 have PD. The survival rate without any neurodegenerative syndrome from diagnosed IRBD patients was 65.2% at 5 years, 26.6% in 10 years, and 7.5% in 14 years. Thus, the diagnose of IRBD appears as a promising tool to assist in early diagnosis of PD.

Besides being present in the premotor phase of PD, RBD also follows the progression of motor symptoms of this disease. A study evaluating 57 patients newly diagnosed with PD and still without the use of DAergic therapy showed that 30% (17 patients) of the individuals had symptoms of RBD [227]. Other studies that investigated the presence of RBD in PD patients treated with DAergic drugs have found prevalence values ranging between 46% and 58% [228, 229]. Gjerstad and colleagues [230] evaluated the presence or absence of RBD in 231 patients with PD reexamining them after 4 and 8 years. Their data exhibited an elevation in the RBD frequency in these patients, from 14.6% to 27%, during the study period. This increase was associated with higher doses of L-DOPA and less Parkinsonism, firming the role of DAergic therapy in the onset of sleep-related disorders.

Although other studies have evaluated melatonin effect in improving sleep quality using subjective scales, only one study investigated the effect in patients diagnosed with RBD [206, 231]. Kunz and Bes studied six patients diagnosed with RBD who received treatment for 6 weeks with 3 mg of melatonin daily, 30 minutes before bedtime. As a result, a dramatic improvement in the symptoms of RBD in 5 patients within a week was observed and this improvement remained after discontinuation of treatment for weeks or months. Polysomnography performed 6 months later revealed that several parameters of REM sleep were normalized.

Insomnia is one of the most common sleep disorders in PD patients and its emergence may be related to uncontrolled motor symptoms, nocturia, depression, and circadian cycle disruption [218]. In a recent study, 689 patients with PD were submitted to a questionnaire in order to evaluate the quality of sleep. This study showed that the total number of patients who presented chronic inability to sleep was 36.9%; 18% had difficulty in initiating sleep, 81.54% interrupted sleep, 40.4% early morning awakenings, and 38.5% nonrestorative sleep [232].

Given the importance of the overnight motor symptoms in PD in the emergence of insomnia, treatments with DAergic drugs in order to reduce the night Parkinsonism have exhibited good efficacy. In this regard, studies assessing the benefits of slow release formulations of ropinirole, rotigotine, or L-DOPA showed an improvement in the sleep quality of patients with PD [233–235]. Several drugs are currently approved for the treatment of insomnia; however, only eszopiclone, an agonist of GABA_A_ receptors, and doxepin, a tricyclic antidepressant, showed some efficacy in the treatment of insomnia in PD patients [221].

The role of melatonin in PD patients who suffer from insomnia is controversial. Although some studies have shown that treatment with different doses of melatonin leads to an improvement in the sleep quality, assessed subjectively by questionnaires or through polysomnography tests [206, 231], other studies show that the light therapy was beneficial for these patients [236]. PD patients exposed to a light intensity of 10000 lux (90 min/6 times a week) obtained an improvement in the insomnia, but there was a worsening in the questionnaires related to the symptoms of PD [237]. Other sleep disorders such as obstructive sleep apnoea, restless legs syndrome, and periodic leg movements during sleep are also often present in PD patients [218], but there is no specific study that associates these disorders with the modulation of the melatoninergic system.

As noted, even though progress has been made towards the characterization of sleep disorders during PD progression and the possible relationship with the melatoninergic system, more studies are needed for the development of effective therapeutic approaches that improve the quality of life of patients and caregivers.

#### 6.2.2. Cognitive Deficits

Prevalence of cognitive deficits in PD patients has been poorly investigated [238]. A study carried out in the UK revealed that 36% of PD patients had cognitive impairment at least in one of three cognitive tasks performed: Minimental State Examination, a pattern recognition task, and the tower of London task [239]. Another report comparing untreated PD with controls found twofold increase in the proportion of cognitive dysfunction. Nevertheless, the lack of criteria for diagnosing cognitive impairment in PD patients makes these studies difficult to reproduce and compare [238].

Cognitive deficits detected in PD differ from individual cognitive impairment to minimal cognitive dysfunction to full-blown dementia like, being divided into mild cognitive impairment (MCI) or severe, leading to the dementia diagnosis (PD dementia (PDD)). This nonmotor symptom of PD may result from the natural course of the disease or could be associated with treatment interventions, such as adverse effects of drugs and deep brain stimulation (DBS). Executive function, memory, and visuospatial deficits are the three cognitive impairments more commonly detected in PD [238].

Executive function comprises essential abilities related to the decision-making process, such as retrieval from declarative memory, management of information in working memory, and cognitive flexibility and planning. Some studies have observed that executive dysfunction is higher during early phase of PD and also that this impairment is a predisposing factor for greater severity of PD [240]. Also, memory deficits, working memory, short-term memory, visual short-term memory, declarative memory, and recognition memory are impaired in PD patients [238]. However, long-term and verbal short-term memory are not altered and explicit memory was shown to be partially lost [238].

Visuospatial dysfunction is well known in PD. Montse et al. [241] demonstrated that a decrease in spatial orientation functioning in PD may elicit the progression rating of the disease as reflected by three determinants variables found: age, duration of disease, and degree of dementia. Moreover, motor involvement has been suggested as major criterion for reduction of this memory, even in the early stages of the illness.

The pattern of cognitive deficits assessed in patients by cognitive tasks suggests the existence of subgroups, which may reflect regional differences in the underlying neuropathological processes [239]. In this way, grey matter loss in most of the brain regions has been correlated with a decrease of global cognitive score, but not with motor impairment. MCI subjects showed partial atrophy of grey matter in the temporal, parietal, and frontal cortex, bilateral caudal hippocampus, amygdala, and right putamen. Brain atrophy occurs as a consequence of the cell death, which is largely attributed to the formation of Lewy bodies in PDD [242].

There is increasing evidence about the role of melatonin in learning and memory processes (Table 3). Wang et al. [169] revealed a concentration-dependent inhibition of long-term potentiation (LTP) by melatonin in the CA1 dendritic layer of the Schaffer collateral of hippocampal slices in mice. LTP is a process in which the neuronal association is cooperatively and selectively strengthened by elevated synaptic activity through glutamate NMDA and *α*-amino-3-hydroxy-5-methyl-4-isoxazolepropionic acid (AMPA) receptors. Melatonin acts on LTP by modulating the postsynaptic nitric oxide (NO) signalling pathway [243]. Thus, this link between melatonin and LTP implies its role in the memory formation [169]. In addition, plasma melatonin levels decrease during aging, being related to the decline of neurogenesis and probably to cognitive alterations [244]. Despite this, the effect of melatonin on cognitive dysfunctions in PD has been poorly investigated, whereas more attention has been given to cognitive impairment in AD.

Datieva et al. [245] evaluated the effect of melatonin in 30 patients with early and late stages of PD and no significant difference was observed in cognitive autonomic disorders when compared to baseline. Using a MPTP rat model of PD, Capitelli et al. [246] investigated the effects of melatonin pretreatment (50 mg/kg, i.p.) in the animals' performance of two-way active avoidance task. The memory acquisition and retention process were impaired by MPTP, and melatonin was unable to restore this deficit.

The association between melatonin and cognitive dysfunction in PD was further addressed in animal models of PD using the novel object recognition (NOR) [247, 248], Morris water-maze (MWM), and passive avoidance tasks [249]. Agomelatine, an agonist of melatoninergic MT_1_ and MT_2_ receptors and an antagonist of serotonergic 5-HT_2C_ receptors, also elicits positive effects on memory [247]. Its chronobiotic activity on cognitive functions was investigated in the T maze left-right spatial discrimination test showing a more intense effect when administered in the evening and in the NOR exhibiting improvement in the memory independent of the administration period. Involvement of melatoninergic agonist and 5-HT_2C_ antagonist properties could be related to the memory facilitating effect of agomelatine [247]. Neu-P11, a melatoninergic (MT_1_/MT_2_) receptors agonist and serotonin 5-HT_1A/1D_ agonist, was able to enhance the memory in the NOR and against deficit induced by A*β*_(1–42)_ peptide [248].

Considering that sleep problems have been shown to contribute to neuropsychological deficits in otherwise healthy people, the nootropic effect of melatonin has been associated with its positive effect on sleep quality. As mentioned before, there is a high prevalence of sleep disturbances in PD patients, which are improved with melatonin treatment. Meta-analysis demonstrated a significant effect of sleep on global cognitive function, long-term verbal recall, long-term verbal recognition, shifting, updating, and fluid reasoning. Specifically in PD, an association between sleep disorders and memory and executive function deficits was found. However, this outcome should be analysed carefully due to the small number of studies and numerous methodological issues identified in them [250].

Furthermore, the differential role of MT receptors has also been investigated, mainly in the hippocampus, in order to elucidate nootropic effect of modulators in this system (Table 3). MT_1_/MT_2_ double knockout mice displayed ameliorated spatial and reference learning and memory performance in the Barnes maze and the Y-maze tests. These behavioural findings were consistent with the enhancement of LTP and brain p-CREB, p-ERK1/2, and key markers of synaptic activity expression levels [171]. However, other studies observed hippocampus LTP inhibition by melatonin that was blocked by MT receptors antagonist [167] and by selective MT_2_ receptor antagonist, suggesting a mediated effect by MT_2_ receptor [168]. This hypothesis was confirmed using MT_1_ knockout and MT_2_ knockout mice, in which the inhibitory LTP action of melatonin was lost in MT_2_^−/−^ mice, but not in MT_1_^−/−^ mice [169].

This prevalent involvement of MT_2_ receptor in the memory and cognitive functions has been demonstrated in the literature. Larson et al. [170] corroborate the data of Wang et al. [169], demonstrating smaller and decrement LTP in hippocampus slices from MT_2_^−/−^ mice. In addition, MT_2_^−/−^ mice showed learning deficits in the elevated plus-maze test (EPM) in two consecutive days, failing to shorten the transfer latencies to enter a closed arm on the second day. Comai and Gobbi [251] also report that MT_2_^−/−^ mice spent more time in the central platform of EPM, which could be linked to an impaired decision taking and cognitive flexibility.

Thus, these studies suggest an important role for melatoninergic system in memory and cognition function, probably linked to underlying process of LTP by MT_2_ receptor. Nevertheless, due to contrasting results, more studies are required to clarify the role of melatoninergic modulators in LTP, as well as the importance of each MT receptor in this regard. After this, the potential use of melatoninergic modulators as cognitive enhancers, mainly for cognitive impairment linked to sleep disorders in PD and AD, should be considered in clinical and preclinical tests.

#### 6.2.3. Depression

Depression represents one of the most common nonmotor symptoms in PD, occurring in 50–70% of patients [252, 253]. Many other diseases that affect elderly people also have depression as common symptom. However, patients with PD are more susceptible to developing depression than the general elderly population or patients with other chronic and disabling diseases [252]. In a recent study conducted with more than 1400 individuals, depression was found to be more common in female than in male patients and had a bigger prevalence in individuals in the advanced stages of PD or in individuals that developed dementia [254].

Depression is an important feature to determine the quality of life in PD patients, and the caregivers of individuals with PD are also affected by the course of the disease [253]. Therefore, understanding depression in PD is a crescent need among all the professionals involved with this disease.

The aetiology of depression in PD is unclear, but alterations in neurotransmitters (monoaminergic) systems and limbic Lewy body pathology might contribute to this disorder. Besides, other previous pathologies like cerebrovascular disease and neurotrophic changes are also related to depression in PD. There is a hypothesis that claims that the monoaminergic deficits and lesions of frontal-subcortical circuits are the same in the brain of patients with PD and those with depression [118].

The main symptoms of depression are depressed mood, loss of pleasure (anhedonia), and feelings of worthlessness and guilty. The depressive syndrome also includes some somatic symptoms such as loss of appetite, sleep disturbances, psychomotor retardation, and altered facial expression [252]. However, these symptoms could also be present in PD patients without the depressive symptoms and the differentiation between these two situations is a great challenge for clinicians.

There are few studies investigating the course of depression among different stages of PD. One of the most important studies in the field evaluated patients with PD who were also diagnosed with major depression. One year later, the majority of these patients still showed major depression and the risk of dementia or a fast cognitive decline that was associated with major depression [255]. Nevertheless, more long-term studies that associate depression and PD are needed to clarify the predictors and prognostic of these patients.

There are contradictories views about depression as a nonmotor symptom of PD or a risk factor for PD development. Jacob et al. [256] and collaborators findings support the hypothesis that depression is a prodromal symptom of PD and not a risk factor for the disease. In a two-year study, it was observed that the development of PD was more common in individuals who had used antidepressants than among nonusers [257].

Depression and PD share some abnormalities in brain structure. In the SNc, there is a hyperechogenicity and in the brainstem raphe there is a reduced echogenicity. These alterations are common and severe in patients who have both PD and depression, and these changes are usually related to a history of depression before the development of PD [258]. Patients with PD and depression also presented a profound loss of striatal DAT availability and frontal hypoperfusion compared with nondepressed patients [252, 253]. These observations, besides confirming that depression can precede PD, are in agreement with Braak hypothesis of PD, in which the neurodegeneration in regions like the noradrenergic coeruleus-subcoeruleus complex and the serotonergic caudal raphe nucleus occurs before the motor symptoms [259].

Defining the mechanism behind depression in PD is very difficult. When people older than 65 years are affected by depression, there are numerous variables involved such as psychosocial issues, genetic factors, and age-related brain alterations that should be considered. In patients with PD that also develop depression, it is necessary to consider the involvement of factors like monoaminergic disturbances, cerebrovascular diseases, Lewy body pathology, functional changes in the limbic and subcortical circuits, hippocampal atrophy, alterations in neurogenesis and neurotrophic factors, and toxic stress, besides hypercortisolemia and inflammation [252].

The neurotransmitter systems affected in patients with depression and PD involve the noradrenergic, DAergic, and serotonergic pathways. These systems are also related to regulation of mood, and deregulations of these are associated with depression in the general population. Patients with PD and depression were shown to have increased serotonergic neuronal cell loss in the dorsal raphe nucleus and decreased levels of striatal serotonin and its transporter. The disruption of the nigrostriatal DA as well as other DAergic pathways in limbic and frontal circuits, and the loss of DAergic neurons in the ventral tegmental area, are also related to depression in PD. Indeed, the locus coeruleus is affected in early stages of PD and the increase of neurodegeneration in this area is correlated with depressed individuals with PD. Cognitive and mood impairments associated with PD could also be related to cholinergic deficits. Furthermore, new evidence suggests that stress hormones, immunomodulatory mediators, neurotrophic factors, and neuropeptides are also involved in the aetiology of depression in PD [252].

Although depression is highly prevalent among individuals with PD, a proper therapy for this disorder has not been well defined. The options of treatment for depression in PD found in literature are limited by a small sample size, short duration of action, design issues, or incompatible outcome measurements [253]. The classic options for the treatment of depression in PD include selective serotonin reuptake inhibitors (SSRI) (citalopram, sertraline, fluoxetine, and paroxetine); serotonin-noradrenalin reuptake inhibitors (SNRI) (desipramine, amitriptyline, nortriptyline, and doxepin); and some atypical agents as trazodone and nefazodone. These are also examples of the main medications used to treat depression in general population. Pramipexole, pergolide, atomoxetine, and memantine are also used, even though they are not so common. Finally, bupropion, a noradrenaline and DA reuptake inhibitor, is normally used to treat depression in PD patients and has effects under the depressive and motor symptoms [123, 124].

Depression in PD has a heterogeneous aetiology and responsiveness to treatments, and although there are a wide variety of treatment options, not all the patients with depression and PD respond to these drugs. Therefore, new strategies and new targets have been investigated to improve the current therapy and assure a good prospect of quality of life for these patients [124]. Melatonin is one of the substances that show beneficial effects for depression in PD patients.

The potential therapeutic effect of melatonin in mood disorders has been studied recently, because many patients with depression presented disruption in melatonin circadian profile [161, 260]. Preclinical studies demonstrated that melatonin has antidepressant-like effects in several animal models of depression, such as in the forced swimming test [260], tail suspension test [261, 262], chronic mild stress paradigm [263], and nonmotor symptoms of a rodent model of PD [161].

In order to document the association between sleep and mood disorders, different studies in humans have been done demonstrating that the treatment with melatonin improves the total sleep time and decreases depressive symptoms. A new pharmaceutical tool for treating and preventing depression contains an association between a SSRI and melatonin; this association had a markedly positive impact on the sleep-wake rhythm of depressive patients [264]. Circadian disruption is linked to the changes in behaviour and sleep patterns that occur in depression, and this disease is associated with alterations in diurnal rhythm of melatonin output [265].

In the rotenone model of PD, the prolonged treatment with melatonin prevented the appearance of depressive-like behaviour, and this melatonin antidepressant effect was associated with its ability to restore the striatal DA levels [161]. Another study demonstrated that the antidepressant-like effect of melatonin in the tail suspension test could be associated with its direct interaction with DA D_1_ and D_2_ receptors [262]. Furthermore, the antidepressant effect of melatonin may also be attributed to the protection against the loss of DAergic neurons and the modulation of DAergic neurotransmission [161].

Since alterations in circadian rhythms are also implicated in the aetiology of depression and PD, dysfunction in the rhythmicity of circadian cycle is very common among PD patients with depression [161, 265]. Another important fact to be observed about melatonin and depression in PD is that the DAergic neurons in the nigrostriatal pathway are melanocytes, and the melatonin and DA coexist in a functional opposition regulating day/night activities. Thus, it is likely that the exogenous melatonin administration may alter the balance between DA and melatonin. In the study of Bolitho et al. [266], the DAergic treatment for PD profoundly increased the secretion of melatonin, regulating the circadian cycle and sleep timing.

Considering the relevance of circadian cycle in the pathogenesis of depression and PD, a new approach has been proposed for the treatment of this disorder using the melatonin analogous agomelatine. This drug is associated with the resynchronization of circadian rhythms and was shown to have antidepressant effect in humans.

Overall, from these limited results in this field, it appears that melatonin and melatonin analogous might be particularly useful to improve the depression in PD, but further studies are needed for the development of effective therapeutic approaches that improve the quality of life of patients and caregivers.

#### 6.2.4. Anxiety

Despite the high prevalence of anxiety in PD patients, this issue has only recently attracted scientific attention. Anxiety disorder presents itself as the second mood disturbance most common in PD, with major impact on daily functioning and quality of life in patients [267].

Prevalence of anxiety in PD patients depends on the study sample features and methodology used to assess this nonmotor symptom [268]. Many studies suggest that approximately 20–50% of PD patients experience clinically relevant symptoms of anxiety [269, 270]. Generalized anxiety disorder (GAD), social phobias, panic attacks, and anxiety disorder Not Otherwise Specified (NOS) are the anxiety syndromes more often reported in PD when using the Diagnostic and Statistical Manual (DSM) of mental disorders criteria for the assessment [271]. Nevertheless, DSM has questionable validity to assess anxiety in PD, since symptoms in PD patients are atypical and therefore are classified as NOS [272]. Recently, Dissanayaka et al. [273] recommended the use of Parkinson's Anxiety Scale (PAS) and Geriatric Anxiety Inventory (GAI) in PD without dementia in order to present satisfactory psychometric features.

Anxiety is a nonmotor symptom of PD that can precede the onset of motor symptoms or develop after the diagnosis of the disease [268]. In order to understand the risk factors linked to anxiety in PD, Broen et al. [274] delineated a model using PD-specific and nonspecific factors. Previous history and the severity of depressive symptoms were considered nonspecific risk factors, which showed direct effect on anxiety. On the other hand, motor fluctuations and disease-related decline in daily activities were considered PD-specific factors and display less influence in the model.

Rutten et al. [275] concluded in their study that anxiety in PD involves affective and somatic symptom dimensions. In fact, several authors have associated anxiety disorders with severe gait problems [270], dyskinesias/fluctuations [270, 271], freezing [276], and depression [267, 270, 274, 276]. Furthermore, depression, urinary disorders, and sleep disruption were found as factors most likely to influence anxiety in PD [272].

Neurobiological alterations related to anxiety in PD are based on two theories [268, 269]. The reactive theory is associated with the diagnostic of PD and therefore with the DAergic lesion and motor impairment [271, 276]. DAergic involvement is supported by positive relationship between anxiety and motor fluctuations and withdrawal of DAergic medication or DBS [111]. Additionally, the mesolimbic DAergic pathway, in which neurodegeneration is observed in PD, sends projections to the amygdala, a key brain structure for the production and regulation of anxiety [277].

In contrast, psychological symptoms could result from early neurochemical changes; hence, epidemiologic observations demonstrate that PD patients have greater predisposition to anxiety disorders before the diagnosis [256]. Thus, anxiety may be a premotor symptom of PD linked with other neurotransmitters systems such as noradrenergic and serotonergic systems [269, 278]. Braak staging scheme for PD suggests early neurodegeneration of the raphe nucleus and the locus coeruleus, structures with abundance in serotonin and noradrenaline, respectively. Serotonin depletion results in an increased anxiety in PD [223], whereas noradrenaline reduction leads to a high frequency of anxiety in PD [279]. Additionally, other neurotransmitter systems such as gamma-aminobutyric acid (GABA) and glutamate neurotransmitters were shown to be altered in PD patients and may also influence anxiety [268, 269].

In spite of the data mentioned, the anxiety in PD is underdiagnosed and undertreated. The treatment of anxiety in patients with PD is the same as that in patients without PD, which is similar to depression treatments [269]. Even though depression and anxiety are frequently comorbid [270, 280], they are distinguished from each other, exhibiting distinct trajectories and different longitudinal relationships with demographic, motor, and nonmotor factors that were unique to each disorder [280].

The benzodiazepine (BDZ) midazolam was compared to melatonin on the perioperative effects in a double-blinded, placebo-controlled study [281]. Both treatments were able to decrease anxiety levels and elevate levels of sedation when compared to control subjects. Nevertheless, midazolam, tested at three different doses, induced impairment in the psychomotor skills and performance on the digit-symbol substitution test at all evaluated time points. Melatonin, at a dose of 0.05 mg/kg, was associated with preoperative anxiolysis and sedation without cognitive and psychomotor deficits. Preclinical trials also were carried out with melatonin and BDZ. Guardiola-Lemaître et al. [236] observed that melatonin enhanced anxiolytic-like action of diazepam in mice submitted to the four plates and tail suspension tests.

Anxiolytic effect of melatonin has also been evaluated on several behavioural tests of anxiety (Table 4). The first evidence of its influence on anxiety was obtained in rats by Golus and King [172] in the open field (OF) test. After this, anxiolytic-like activity of melatonin has been observed in several behavioural animal tests, such as light/dark box (LD), elevated plus-maze (EPM), and Vogel tests [173–176, 178–182]. Papp et al. [178] reported the capacity of melatonin in increasing the open-arm exploration at the EPM only in the evening experiment and proving itself inactive in the Vogel and ultrasonic vocalization tests independent of timing [178]. Nonetheless, Millan et al. [177] data do not corroborate this conclusion. The variability found in the results may be related to dose and time of delivery, but there is a consensus that anxiolytic melatonin's effect is strongly time dependent [173, 179, 180] (Table 4).

In relation to clinic findings, Datieva et al. [245] described the effectiveness of melatonin in decreasing the anxiety state on Spielberger's scale without altering motor, cognitive autonomic, and depression levels in patients with early and late stages of PD. Melatonin treatment also presented itself as useful as an anxiolytic in perioperative treatment in nine of the ten studies. In addition, plasma melatonin is diminished at night in patients with panic disorder, which has an increased prevalence in PD patients [282].

Other modulators of melatoninergic system have been investigated on anxiety disorders. Agomelatine, Neu-P11, and UCM765 presented anxiolytic effects in behavioural animal tests independent of the timing of administration [177, 178, 180, 182], without inducing sedation [178]. Clinical data suggest that agomelatine has anxiolytic potential, which proved to be equal or higher than SSRIs and SNRIs for the treatment of GAD, a common anxiety disorder in PD [283]. Case reports suggest a potential of agomelatine as treatment for social anxiety [284] and panic disorders [285]. Kalyn et al. [286] assessed therapeutic efficacy, tolerability, and safety of agomelatine in elderly patients observing positive results in depression, anxiety, and anhedonia indicators. On the other hand, anxiolytic action of UCM765 was shown to be mediated by MT_2_ receptors [182] and anxiety levels did not alter in MT_2_^−/−^ mice in the EPM [251].

Different effects on anxiety found among melatonin and melatoninergic agonists mentioned here suggest the involvement of other neurotransmitter systems. The use of S22153, a melatonin receptor antagonist, was shown to block both melatonin and agomelatine anxiolytic effects in the EPM at night [178], reinforcing the participation of MT receptors in the nocturnal action. However, Millan et al. [177] did not report the same effect, suggesting that the anxiolytic action of agomelatine is more related to 5-HT_2C_ antagonism. Recently, the capacity of MT_2_ and 5-HT_2C_ receptors to assemble into functional heteromers was identified, indicating a potential involvement of these heteromers in agomelatine effect [51]. The maintenance of anxiolytic action of agomelatine in daytime also corroborates the modulation of serotoninergic system. In addition, there are several evidences of the contribution of the GABAergic system to melatonin, agomelatine, and Neu-P11 effects [173, 174, 179, 180].

Melatonin can bind to GABA receptors like BDZ, barbiturates, and GABA, enhancing chloride influx [287]. Furthermore, enhancement or attenuation of GABA_A_ receptor-mediated currents strongly depends on melatonin receptors activation [287]. Indeed, melatonin potentiated GABA-evoked current amplitude in suprachiasmatic nucleus, which express MT_1_ receptors, and decreased in hippocampal CA1 neurons, which express MT_2_ receptors [166]. Pharmacological interventions also elicit the involvement of GABAergic system in the effect of melatonin [173, 174, 236]; and in humans it has been used to reduce or eliminate BDZ administration in elderly patients [251].

Therefore, there are many studies supporting the anxiolytic potential of melatonin and other melatonin receptors agonists, demonstrating the cross talk between melatoninergic, GABAergic, and serotoninergic systems. Nonetheless, its use in PD, a disease with high prevalence of anxiety disturbances, has been underinvestigated in both preclinical and clinical studies.

Although research is at an early stage, the findings reviewed above highlight the sleep, memory, and anxiety- and mood-enhancing properties of melatonin in humans and rodents. Therefore, the performance of additional preclinical and clinical studies to verify the effects of melatonin and new drugs acting on melatoninergic system in the nonmotor symptoms of PD appears to be a promising field.

## 7. Conclusions

This article reviews the recent evidence that the melatonin plays an important role not only as a modulator of light-dark cycle, but fundamentally as an important factor for neuronal plasticity, protection, and survival in the CNS. Possible therapeutic opportunities for neurological disorders through the modulation of melatoninergic system are presented in the expectancy that this review may inspire clinical researchers and foster experimental approaches using melatonin receptors MT_1_ and MT_2_ as therapeutic targets.

Melatonin has anti-inflammatory and antioxidant properties acting as a free-radical scavenger, reducing hydroxyl free radicals, and improving mitochondrial homeostasis. It also regulates the expression of neurotrophins that are directly involved in the survival of dopaminergic neurons and reduces the aggregation of *α*-synuclein restoring deficits in the DAergic system. The unbalance of pineal melatonin synthesis can predispose the organism to inflammatory and neurodegenerative diseases such as PD. The literature reviewed here indicated that PD is associated with impaired brain expression of melatonin and its receptors MT_1_ and MT_2_. Moreover, melatonin administration has demonstrated an outstanding neuroprotective effect in animal models of PD induced by different toxins such as 6-OHDA, MPTP, rotenone, paraquat, and maneb. These studies have shown promising results with the improvement of motor deficits in animal models of PD and notably in nonmotor symptoms such as sleep disorders, anxiety, depression, memory, and cognition dysfunctions that are part of the symptoms commonly experienced by PD patients.

Future research aiming at the development of novel melatonin receptor selective ligands will help to elucidate the neurobiology and physiological role of melatonin as well as its potential as a novel palliative and neuroprotective agent in PD.

## Figures and Tables

**Table 1:** Drugs used for symptomatic treatment of Parkinson's disease.

Drug	Mechanism of action
L-DOPA	DA precursor
L-DOPA + benserazide or carbidopa	DA precursor + peripheral dopa-decarboxylase inhibitor
Bromocriptine, pergolide, pramipexole, ropinirole	DA receptor agonists
Selegiline, rasagiline	Monoamine oxidase B inhibitors
Amantadine	Increase of DA release and glutamate NMDA receptor antagonist
Trihexyphenidyl, benztropine	Muscarinic receptor antagonists
Entacapone, tolcapone	Catechol-O-methyltransferase inhibitors

Adapted from [108].

**Table 2:** Summary of studies presenting neuroprotective effects of melatonin in *in vivo *and *in vitro* models of PD.

Toxin	Subjects	Experimental approach	Main findings	Ref.
6-OHDA	Male Wistar rats	Unilateral injection of 6-OHDA (8 *μ*g) into the right SNc. Treatment with melatonin (1 and 10 mg/kg, i.p.) before apomorphine administration.	Melatonin treatment inhibited apomorphine-induced rotational behaviour.	[131]
	PC12 cells	Preincubation (3 h) with melatonin (10^−7^ and 10^−9^ M). Incubation with 6-OHDA (25, 50, 100, and 250 *μ*M).	Melatonin prevented the loss of cell viability and apoptosis induced by 6-OHDA. Melatonin also protected the reduction of mRNAs of antioxidant enzymes evoked by 6-OHDA.	[132]
	Male Sprague-Dawley rats	Unilateral injection of 6-OHDA (20 *μ*g in 5 *μ*L) into the right striatum. Melatonin (3 and 10 mg/kg, i.p.) was administrated 1 h before and immediately and 1 and 2 h after 6-OHDA injection. After that, the animals received a daily administration of melatonin in the next 3 days.	Melatonin treatment recovered the 6-OHDA-induced changes in striatal MDA and DA levels and TH activity.	[133]
	PC12 cells	Preincubation (3 h) with melatonin 10^−7^ M. Incubation with 25, 50, and 100 *μ*M of 6-OHDA.	Melatonin protected cells from apoptosis and necrotic lesions induced by 6-OHDA.	[134]
	Male Sprague-Dawley rats	Unilateral injection of 6-OHDA (8 *μ*g in 2 *μ*L) into the right SNC. Melatonin treatment (50 ± 7.5 *μ*g/h, s.c.) started immediately after 6-OHDA injection and it was maintained for 7 days.	Melatonin treatment prevented apomorphine-induced rotational behaviour and loss of complex I activity induced by 6-OHDA.	[135]
	Male Wistar rats	Unilateral injection of 6-OHDA into the right striatum (two injections of 12 *μ*g in 1 *μ*g of saline). Posttreatment (1 h) with melatonin (2, 5, 10, and 25 mg/kg, i.p.), daily for 7 days.	Melatonin prevented 6-OHDA-induced depletion of striatal DA and serotonin levels. Melatonin blocked the apomorphine-induced rotational behaviour.	[136]
	SK-N-SH cells	Preincubation (1 h) with melatonin (0.1, 0.5, 1.0, and 2.0 mM). Incubation with 6-OHDA (100 *μ*M) for 24 h.	Melatonin protected against 6-OHDA-induced loss of cellular viability and increased activity of c-Jun-N terminal kinase signalling cascade.	[137]
	Male Sprague-Dawley rats	Unilateral injection of 6-OHDA (8.75 *μ*g) into striatum. After lesion, animals received melatonin (0.4 or 4 *μ*g/mL) in drinking water for 10 weeks.	Melatonin 4 *μ*g/mL recovered motor deficits and normalized TH immunoreactivity and GDNF mRNA levels.	[138]
	Female Sprague-Dawley rats	Pretreatment with melatonin (0.5 mg/kg, i.p.) for 7 days. On day 8, animals received an unilateral injection of 6-OHDA (8 *μ*g) into the lateral striatum.	Melatonin treatment prevented motor deficits (observed in the apomorphine-induced rotational behaviour, staircase test, disengage time, stepping test, initiation time, and postural balance test) induced by 6-OHDA administration.	[139]
	Male Wistar rats	Unilateral injection of 6-OHDA (8 *μ*g) into the right medial forebrain bundle (MFB). Melatonin treatment (10 mg/kg, p.o.) began four days after 6-OHDA injection and continued for 30 days.	Melatonin treatment improved motor performance without evoking dyskinesia. Melatonin also protected TH-positive neurons and neuronal ultrastructure of striatum.	[140]
	Male Wistar rats	Unilateral injection of 6-OHDA (12 *μ*g) into the right MFB. Melatonin (10 mg/kg/day, i.p.) was administrated 23 days before and 7 days after (pre- and posttreatment) or only 7 days after (posttreatment) the injection of 6-OHDA.	Melatonin decreased COX and caspase-3 activity and PGE2 levels and increased Bcl-2 levels that have been altered by 6-OHDA injection. Melatonin also prevented the loss of DAergic neurons in SNc.	[141]
	Male Sprague-Dawley rats	Unilateral injection of 6-OHDA (20 *μ*g in 5 *μ*L) into the right striatum. Melatonin (3 and 10 mg/kg, i.p.) was administrated 1 h before and immediately and 1 and 2 h after 6-OHDA injection. After that, the animals received a daily administration of melatonin in the next 3 days.	Melatonin treatment reduced motor deficits and protected against 6-OHDA-induced loss of DAergic neurons in SNc and in dorsolateral striatum.	[142]
	Male Wistar rats	Unilateral injection of 6-OHDA (12 *μ*g) into the right MFB. Melatonin (10 mg/kg/day) was administrated 23 days before and 7 days after (pre- and posttreatment) or only 7 days after (posttreatment) the injection of 6-OHDA.	Melatonin treatment protected against the 7-OHDA-induced loss of DAergic neurons, increased antioxidant enzyme activities (SOD, catalase and GPx), and decreased lipid peroxidation. The pretreatment with melatonin was more effective in protecting against the 6-OHDA-induced deficits.	[143]

MPP^+^	Female and male Sprague-Dawley rats	Pretreatment (30 min) with melatonin (10 mg/kg, i.p.). Unilateral injection of MPP^+^ (7.4 *μ*g) into the right SNc. Animals were subjected to an acute or chronic posttreatment with melatonin.	Melatonin treatment reduced lipid peroxidation and protected against DAergic neuronal loss induced by MPP^+^.	[144]
	Hepatic mitochondria and striatal synaptosomes	Preincubation with melatonin (10^−6^ to 10^−3^ M). Incubation with MPP^+^ (10^−6^ to 10^−3^ M).	Melatonin prevented the inhibition of complex I induced by MPP^+^.	[145]
	Male Wistar rats	Unilateral injection of MPP^+^ (0.1 *μ*mol) into the right striatum. Melatonin (10 mg/kg, i.p.) was administrated 1 h before and 1, 3, and 5 h after MPP^+^ administration.	Melatonin reduced the MPP^+^-induced DAergic toxicity and recovered the GSH levels.	[146]
	SH-SY5Y cells	Preincubation (4 h) with melatonin (200 *μ*M). Incubation (72 h) with MPP^+^ (1 mM).	Melatonin reduced MPP^+^-induced mitochondrial DNA oxidative damage, accumulation of oxygen free radicals, generation of mitochondrial membrane potential collapse, and cell death.	[147]
	Cerebellar granule cells	Coincubation with MPP^+^ (200 *μ*M) and melatonin (1 mM) at the same time.	Melatonin protected cell viability and prevented apoptosis. Melatonin also reduced cdk5 expression and the cleavage of cdk5-35 to cdk5-25 induced by MPP^+^.	[148]
	SK-N-SH cells	Preincubation (1 h) with melatonin (1 mM). Incubation with MPP^+^ (0.1 mM).	Melatonin prevented the MPP^+^-induced phosphorylation of c-Jun, activation of caspase-3, DNA fragmentation factor 45 (DFF45), and DNA fragmentation.	[149]
	Adult Wistar rats	Injection of 1 *μ*L of 50 mM MPP^+^ into the right striatum. Melatonin (10 mg/mL, i.p.) was administrated 0, 1, 2, 3, 4, 24, 48, and 72 h after MPP^+^ injection.	Melatonin protected DAergic neurons from apoptosis induced by MPP^+^. Melatonin recovered mRNA and protein expression of fibroblast growth factor 9 that was reduced by MPP^+^ injection.	[150]

MPTP	C57BL/6 mice	Single injection of MPTP (20 mg/kg, s.c.). Melatonin (10 mg/kg i.p.) was administrated 30 min prior to and every hour (for 3 h) after MPTP injection.	Melatonin treatment prevented MPTP-induced lipid peroxidation and TH-positive neurons loss in striatum.	[151]
	Male C57BL/6 mice	Single injection of MPTP (15 mg/kg, s.c.). Melatonin (5 or 10 mg/kg i.p.), deprenyl (0.37 mg/kg), or deprenyl plus melatonin (0.37 mg/kg and 5 or 10 mg/kg) was administrated 30 min prior to MPTP.	Melatonin was able to protect the mitochondrial complex I activity and the oxidative damage in nigrostriatal neurons. Melatonin treatment also potentiates the protective effect of deprenyl on DA levels and TH activity.	[152]
	Male C57BL/6 mice	Four injections of MPTP (15 mg/kg, s.c.) with intervals of 2 h. After 24 h, the animals received three additional injections with the same dose and intervals. Melatonin (20 mg/kg s.c.) was administrated 1 h before the first injection of MPTP.	Melatonin treatment prevented the MPTP-induced mitochondrial iNOS in striatum and SNc. Melatonin also protected complex I activity and inhibited lipid peroxidation.	[153]
	Rat astrocytoma cell	Preincubation with melatonin (50, 100, and 200 *μ*M). Incubation with MPTP (400 *μ*M).	Melatonin decreased the MPTP-induced oxidative and nitrosative stress, intracellular calcium, and activation of P-p38 MAPK. Melatonin also normalized the levels of inflammatory proteins, mRNA of proinflammatory cytokines, and NF-*κ*B.	[154]
	Male C57BL/6 mice	Ten injections of MPTP (15 mg/kg, i.p.) during 5 weeks (2 injections a week). Melatonin (5 mg/kg, i.p.) was administered 1 week before, 5 weeks during, and 12 weeks after MPTP treatment.	Melatonin recovered mitochondrial respiration, ATP production, and antioxidant enzyme levels. Melatonin also protected against MPTP-induced DAergic neurons loss and locomotor activity deficits.	[155]
	Male Swiss mice	Four injections of MPTP (20 mg/kg, i.p.) with 2 h between them. Eight days after MPTP injections, animals received L-DOPA/carbidopa (100/10 mg/kg/twice/day, p.o.) and/or melatonin (5 or 10 mg/kg/day, p.o.) for 8 weeks.	Melatonin treatment recovered motor performance, striatal DA level, GSH, and antioxidant enzyme activities and reduced lipid peroxidation. Melatonin also improved the motor response to L-DOPA.	[156]
	Male BALB/c mice	MPTP (30 mg/kg, i.p.) was administrated in two injections (16 h apart). Melatonin treatment (10, 20, and 30 mg/kg, i.p.) 30 min before MPTP administration, followed by four doses of melatonin, at every 10 h.	Melatonin protected against the MPTP-induced TH-positive neurons loss in SNc and enhanced the effects of L-DOPA treatment.	[157]
	Embryos of zebrafish	Incubation with MPTP (600 *μ*M). Incubation with melatonin (0.2 and 1.0 *μ*M) at the same time or after the MPTP treatment.	Melatonin recovered motor behaviour of the embryos. Melatonin also restored gene expression and normal function of parkin/PINK1/DJ-1/MUL1 loop.	[158]

Rotenone	*Drosophila melanogaster*	Melatonin (5 mM) and/or rotenone (125 *μ*M) were added to the feeding medium for 7 days.	Melatonin treatment prevented motor deficits and neuronal loss.	[159]
	Male Sprague-Dawley rats	Rotenone injection (6 *μ*g in 1 *μ*L) into the right SN. Melatonin (10, 20, and 30 mg/kg, i.p.) was administrated 30 min after rotenone injection and was given every 12 h for 4 days.	Melatonin reduced the levels of hydroxyl radicals in the isolated mitochondria and protected GSH levels and antioxidant enzymes activities in SN that were changed by rotenone injection.	[160]
	Male Wistar rats	Rotenone injection (2.5 mg/kg, i.p.) for 10 days. Melatonin (10 mg/kg, i.p.) was administrated for 28 days after the rotenone injection.	Melatonin treatment protect TH-positive neurons in SNc and striatal levels of dopamine. Melatonin also inhibit the rotenone-induced depressant-like effect.	[161]
	Male Sprague- Dawley rats	Three injections of rotenone (4.0 *μ*g in 2.0 *μ*L/site) at three points along its rostrocaudal axis. Animals received melatonin (4.0 *μ*g/mL) in drinking water, one week before and nine weeks after rotenone injections.	Melatonin treatment protected TH-positive neurons in striatum and SNc. Melatonin also inhibited the rotenone-induced loss in dopamine of SNc and apomorphine-induced rotations.	[162]

Maneb	PC12 cells	Incubation (2 h) with melatonin (1 nM) and/or maneb (1 *μ*g/mL).	Melatonin prevented the maneb-induced disruption of the mitochondrial transmembrane potential, activation of caspase-3/7, loss in cell viability, and aggregation of *α*-synuclein.	[163]

Maneb plus paraquat	Male Swiss mice	Treatment with melatonin (30 mg/kg/day, i.p.) for 9 weeks. Treatment with maneb (30 mg/kg, i.p.) plus paraquat (10 mg/kg, i.p.) twice a week, for 9 weeks, 2 hours after melatonin injection.	Melatonin treatment protected the maneb/paraquat-induced lipid peroxidation, TH-positive neurons degeneration, increased nitrite content and mRNA levels of cytochrome P-450 2E1, GSTA4-4 activity, and increased levels of glutathione-S-transferase, P-p53, Bax, and caspase-9.	[164]

Lentiviral vector	Male Sprague-Dawley rats	Injection with lentiviral vectors encoding A30P mutant human *α*-synuclein (lenti-A30P) into the right SNc. Melatonin treatment (10 mg/kg/day, i.p.) 2 days before and 8 weeks after the injection of lenti-A30P.	Melatonin treatment prevented the loss of TH-positive neurons induced by injection of lenti-A30P.	[165]

6-OHDA: 6-hydroxydopamine; COX: cyclooxygenase; DA: dopamine; GDNF: glial cell-derived neurotrophic factor; GPx: glutathione peroxidase; GSH: reduced glutathione; GSTA4-4: glutathione S-transferase alpha 4; i.p.: intraperitoneal; iNOS: inducible nitric oxide synthase; MAPK: mitogen-activated protein kinases; MDA: malondialdehyde; MPP^+^: 1-methyl-4-phenylpyridinium; MFB: medial forebrain bundle; MPTP: 1-methyl-4-phenyl,1-1,2,3,6-tetrahydropyridine; NF-*κ*B: nuclear factor-*κ*B; PGE2: prostaglandins E2; s.c.: subcutaneous; SNc: substantia nigra pars compacta; SOD: superoxide dismutase; TH: tyrosine hydroxylase.

**Table 3:** Summary of studies investigating the role of melatonin and its receptors in LTP and learning and memory processes.

Experimental approach	LTP	Main findings	MT receptor involved	Ref.
Melatonin 1 nM	Inhibits	Melatonin inhibits GABA_A_ via MT_2_.	MT_2_	[166]
Melatonin 0.1 to 2.0 mM	Inhibits	Luzindole, an antagonist of MT receptors, blocks the inhibitory effect.	MT	[167]
Melatonin 100 *μ*M	Inhibits	BMNEP, a specific ligand of the MT_2_ receptors, mimics the inhibitory action.	MT_2_	[168]
Melatonin 0.1 nM to 100 *μ*M; MT_1_^−/−^ mice and MT_2_^−/−^ mice	Inhibits	Melatonin inhibitory action is prevented by luzindole and 2-propionamidotetraline, an MT_2_ antagonist. MT_2_^−/−^ mice lost inhibitory effect on LTP, but not MT_1_^−/−^ mice.	MT_2_	[169]
MT_2_^−/−^ mice	Inhibits	Slices from MT_2_^−/−^ mice exhibited smaller and decrement LTP compared to wild type mice. MT_2_^−/−^ mice showed impairment in the EPM on 2 consecutive days.	MT_2_	[170]
MT_1_/MT_2_^−/−^ mice	Enhances	MT_1_/MT_2_^−/−^ mice demonstrated improvement in cognitive performance in the Barnes- and Y-maze tests.	MT_1_/MT_2_	[171]

**Table 4:** Animal studies addressing the effects of melatonin on anxiety-like responses.

Test	Gender, species, stain	Melatonin treatment	Effects	Ref.
OFT	Male, rat, Wistar	1 mg/kg, i.p.	Anxiolytic	[172]
EPM	Male, rat, Wistar	1–20 mg/kg, i.p. at 12:00 h or 18:00 h	Anxiolytic during the dark phase	[173]
FET, LDT	Male, mouse	0.5–5 mg/kg, i.p.	Anxiolytic	[174]
Vogel test	Male, rat, Wistar	0.1–2.0 mg/kg, i.p.	Anxiolytic	[175]
FET, LDT	Male, mouse, C3H/He	5–25 mg/kg, p.o.	Anxiolytic	[176]
EPM, Vogel test, USV, social interaction test (SIT)	Male, rat, Wistar	2.5–80 mg/kg, i.p.	Anxiogenic at 80 mg/kg on the SIT No effect on the other tests	[177]
EPM, Vogel test, USV	Male, rat	10–75 mg/kg 2 h before 2 h after the dark phase	Anxiolytic on the EPM 2 h after the dark phase No effect on the Vogel test and USV	[178]
Vogel test	Male, rat, Wistar AF	20–80 mg/kg, i.p. at 17:00 to 20:00 h	Anxiolytic	[179]
EPM	Male, rat, Sprague-Dawley	50 mg/kg, i.p. in the morning and afternoon	Anxiolytic in the afternoon	[180]
EPM, OFT	Male and female, rat, Wistar	4 mg/kg, s.c. at 16:00 h for 8 weeks	Anxiolytic Gender-dependent	[181]
EPM, OFT, NSF	Male, rats, Sprague-Dawley	20 mg/kg, s.c.	Anxiolytic on the EPM and NSF No effect on the OFT	[182]

EPM: elevated plus-maze test; FET: free exploratory test; LDT: light/dark box test; NSF: novelty-suppressed feeding test; OFT: open field test; USV: ultrasonic vocalization.
